# Patient-reported outcomes in Philadelphia chromosome-positive acute lymphoblastic leukemia patients treated with ponatinib or imatinib: results from the PhALLCON trial

**DOI:** 10.1038/s41375-025-02608-4

**Published:** 2025-04-16

**Authors:** Ajibade Ashaye, Ling Shi, Ibrahim Aldoss, Pau Montesinos, Pankit Vachhani, Vanderson Rocha, Cristina Papayannidis, Jessica T. Leonard, Maria R. Baer, Jose-Maria Ribera, James McCloskey, Jianxiang Wang, Sujun Gao, Deepali Rane, Shien Guo

**Affiliations:** 1 Takeda Development Center Americas, Inc., Cambridge, MA USA; 2 Evidera Inc., Bethesda, MD USA; 3 City of Hope National Medical Center, Duarte, CA USA; 4 Hospital Universitari i Politècnic La Fe, Valencia, Spain; 5 University of Alabama at Birmingham, Birmingham, AL USA; 6 Instituto do Câncer do Estado de São Paulo, University of São Paulo, São Paulo, Brazil; 7 IRCCS Azienda Ospedaliero-Universitaria di Bologna, Istituto di Ematologia “L. e A. Seràgnoli”, Bologna, Italy; 8 Oregon Health and Science University, Portland, OR USA; 9 University of Maryland Marlene and Stewart Greenebaum Comprehensive Cancer Center, Baltimore, MD USA; 10 ICO—Hospital Germans Trias i Pujol, Josep Carreras Leukaemia Research Institute, Badalona, Spain; 11 Hackensack University Medical Center, Hackensack, NJ USA; 12 Institute of Hematology & Blood Diseases Hospital of CAMS & PUMC, Tianjin, China; 13 The First Hospital of Jilin University, Changchun, China

**Keywords:** Quality of life, Randomized controlled trials

## Abstract

In the Phase 3 PhALLCON trial (NCT03589326), ponatinib demonstrated superior efficacy and comparable safety profile versus imatinib in adults with newly diagnosed Philadelphia chromosome-positive (Ph+) acute lymphoblastic leukemia (ALL). Here we report patient-reported outcomes (PRO) from PhALLCON assessed as exploratory endpoints using the Functional Assessment of Cancer Therapy-Leukemia (FACT-Leu) and EQ-5D-5L. Primary PRO domains included FACT-G physical well-being, FACT-Leu subscale (FACT-LeuS), Trial Outcome Index (TOI), FACT-Leu total score, FACT-G total score, and EQ-5D visual analogue scale. Differences in least-squares mean score changes from baseline to the end of induction (EOI)/consolidation (EOC) and time to confirmed improvement/deterioration were analyzed. Overall treatment tolerability was assessed using the FACT-GP5. Analyses included 238 patients (ponatinib 159, imatinib 79) with ≥1 PRO assessment. Least-squares mean changes from baseline favored ponatinib, with significant and meaningful differences in FACT-LeuS, TOI, and FACT-Leu total score at EOI and across the primary domains except for FACT-LeuS at EOC. Median time to confirmed improvement was shorter with ponatinib versus imatinib for key measures. Ponatinib-treated patients tended to report being less bothered by treatment side effects as assessed by FACT-GP5. These findings highlight ponatinib’s potentially favorable impact on health-related quality of life, supporting its use as frontline treatment for Ph+ ALL.

## Introduction

Acute lymphoblastic leukemia (ALL) is a malignant disorder characterized by the uncontrolled proliferation of lymphoid progenitor cells and is the most common malignancy of childhood. In the United States, the estimated incidence of ALL is 6550 new cases in 2024, accounting for 10% of new leukemia cases [1]. The 5-year survival rate for ALL decreases with age at diagnosis, from 39% for patients diagnosed at age 45–54 to 18% for those diagnosed at age ≥65 [2]. Philadelphia chromosome positive (Ph+) ALL is a subtype of ALL harboring the *BCR::ABL1* fusion gene, which encodes the BCR::ABL1 oncoprotein, a constitutively activated tyrosine kinase [3]. Ph+ ALL accounts for approximately 25% of adult and 3%–5% of pediatric ALL cases [4, 5].

The current standard of care for patients with newly diagnosed Ph+ ALL is chemotherapy combined with a tyrosine kinase inhibitor (TKI), which blocks the BCR::ABL1 oncoprotein [6]. These regimens have been shown to significantly improve response rate and survival outcomes compared with traditional chemotherapy [7]. However, treatment resistance to the first-generation (imatinib) or second-generation (dasatinib and nilotinib) TKIs often develops due to the emergence of BCR::ABL1 kinase domain mutations, particularly the T315I mutation, limiting the efficacy of these targeted therapies [8–10].

Ponatinib is a third-generation, potent, pan-BCR::ABL1 TKI for treating Ph+ leukemia, with activity against tumors expressing BCR::ABL1, including those with the T315I mutation [11, 12]. The efficacy and safety of ponatinib in newly diagnosed Ph+ ALL were demonstrated in the phase 3, randomized ponatinib-3001 trial (referred herein as PhALLCON). The final analysis of the primary efficacy endpoint in PhALLCON showed a significantly higher rate of minimal residual disease (MRD)-negative complete remission (CR) at the end of induction with ponatinib than with imatinib (34% vs. 17%) when combined with reduced-intensity chemotherapy [13]. The safety profile of ponatinib was comparable to that of imatinib. At the data cutoff of August 2022, median event-free survival was reached in the imatinib arm, but not in the ponatinib arm, with a trend favoring ponatinib (hazard ratio [HR] 0.65; 95% confidence interval [CI]: 0.39, 1.10). Based on these results, ponatinib was recently granted accelerated approval for the treatment of newly diagnosed Ph+ ALL in adults in combination with chemotherapy by the United States Food and Drug Administration [14]. Data on the impact of ponatinib on health-related quality of life (HRQoL) are needed to fully understand the efficacy and tolerability of the treatment.

Patient-report outcome (PRO) data provide insight into patient experience with treatment and have been recommended to support the clinical benefits of new cancer treatments, particularly when mature survival data are not yet available at the time of regulatory decision [15–17]. This post hoc exploratory analysis aimed to evaluate the effect of ponatinib compared with imatinib on PROs in PhALLCON.

## Methods

### Study design and participants

The phase 3, randomized, open-label, multicenter PhALLCON trial (NCT03589326) enrolled adults (age ≥18 years) with newly diagnosed Ph+ or *BCR::ABL1*-positive ALL, as defined by the National Comprehensive Cancer Network guidelines [18]. Details of the trial have been reported previously [13]. The trial was conducted in accordance with the International Council for Harmonisation (ICH) Guideline for Good Clinical Practice and the Declaration of Helsinki. The study protocol was approved by the independent institutional review board at each study site before trial initiation. All patients provided written informed consent.

Eligible patients were randomized 2:1 to receive ponatinib (30 mg once daily) or imatinib (600 mg once daily) orally in combination with reduced-intensity chemotherapy through induction (cycles 1–3), consolidation (cycles 4–9), and maintenance (cycles 10–20) phases in 28-day cycles. Randomization was stratified by age (18 to <45, 45 to <60, and ≥60 years). After cycle 20, patients received single-agent ponatinib or imatinib. Patients remained on study treatment until death, relapse from CR, disease progression, unacceptable toxicity, consent withdrawal, or receipt of hematopoietic stem cell transplantation (HSCT) or alternative therapy. Patients who did not achieve MRD-negative (*BCR*::*ABL1*^*IS*^ ≤ 0.01% [MR4] [19]) CR at the end of induction discontinued study treatment. Those who had either CR or MRD-negative status at the end of induction could remain on study treatment at the investigator’s discretion.

The primary endpoint was the percentage of patients achieving MRD-negative CR for 4 weeks at the end of induction. The effect of ponatinib on PRO endpoints was compared with that of imatinib as an exploratory objective and PRO data were analyzed post hoc.

### PRO assessments

Patients completed the Functional Assessment of Cancer Therapy–Leukemia (FACT-Leu) and EQ-5D-5L by filling out electronic and/or paper forms at screening/baseline; cycle 1 day 1 (C1D1), C4D1, C7D1, C10D1, C13D1, C16D1, C19D1, and C21D1, and at least every 6 cycles thereafter (e.g., C27D1, C33D1); and at the end of treatment (EOT) (Supplementary Table 1). The two instruments were selected as both have been validated with acceptable psychometric properties in patients with leukemia or other cancers [20–22] and are commonly used to assess HRQoL in acute leukemia trials [23–26].

The FACT-Leu comprises a FACT-General (FACT-G) scale assessing physical well-being (PWB), social/family well-being (SWB), emotional well-being (EWB), and functional well-being (FWB), and an “additional concerns” subscale (FACT-LeuS) with leukemia-specific items measuring symptoms such as fever, pain, chills, night sweats, lumps or swelling, bleeding, bruise, fatigue, emotional disturbance, and feeling of isolation (Supplementary Table 2). Three summary scores, FACT-Leu Trial Outcome Index (TOI), FACT-G total score, and FACT-Leu total score, are calculated from different combinations of the FACT-G domain and FACT-LeuS scores. The FACT-G PWB domain includes the item GP5 (“I am bothered by side effects of treatment”), which has been recommended as a summary measure of overall treatment tolerability in clinical studies of cancer treatments [16]. The scoring of each domain and summary score, including the handling of missing items, followed the guidelines outlined in the developer’s published manual [27].

The EQ-5D-5L comprises mobility, self-care, pain/discomfort, usual activities, and anxiety/depression dimensions, each with 5 levels of severity [28]. A weighted health utility index (HUI) was calculated by crosswalk method mapping to the EQ-5D-3L UK and US value sets [29, 30]. The EQ-5D-3L HUI based on the UK population weights ranges from −0.594 to 1.0, with a score of 0 indicating death, 1.00 indicating “full health,” and negative scores reflecting states perceived to be worse than death. The HUI based on the US value set ranges from −0.109 to 1.0. The EQ-5D-5L also contains a visual analogue scale (EQ-VAS) ranging from 0 (worst imaginable health state) to 100 (best imaginable health state).

FACT-G PWB, FACT-LeuS, FACT-Leu TOI, FACT-G total score, FACT-Leu total score, and EQ-VAS were selected as primary domains (scales) of interest prior to the initiation of PRO analyses based on their clinical relevance and importance to the target population [26, 31]. The other PRO domains were assessed as secondary domains of interest. The interpretation of the meaningfulness of changes from baseline in each PRO domain at the end of induction and consolidation was based on published minimal important difference (MID) thresholds for within-group change, between-group difference, and within-patient change (responder definition [RD]) [32–37] (Supplementary Table 3).

PRO results are reported according to the CONSORT-PRO guideline [38].

### Statistical analyses

Data cutoff date was August 12, 2022. The proportions of the intent-to-treat (ITT) patients expected and not expected to complete the PRO assessments along with the corresponding reasons at each scheduled assessment visit during the treatment phase were descriptively summarized by treatment arm. The completion rate, calculated as the number of ITT patients with an evaluable PRO assessment divided by the number of ITT patients expected to have a PRO assessment at each visit, was summarized for each arm.

ITT Patients with ≥1 PRO assessment were included in the PRO analysis (PRO-evaluable population). Patient demographics, clinical characteristics, and baseline PRO scores were summarized descriptively. Mean baseline PRO scores were also compared with the normative general population data [39–41] to contextualize the average HRQoL level of the analysis population at baseline. Observed PRO score changes from baseline were descriptively summarized for post-baseline visits with a sample size of ≥10 for each arm. Distributions of FACT-GP5 responses and response level changes from baseline were descriptively summarized by treatment arm at each visit.

Comparative assessment of PRO endpoints between ponatinib and imatinib was conducted according to the guidance of the ICH E9(R1) estimand framework [42]. In the current analyses, death and treatment discontinuation due to any other cause (e.g., not achieving MRD-negative CR, relapse from CR or progressive disease, receipt of HSCT or alternative therapy, or unacceptable treatment toxicity) were the relevant intercurrent events (ICEs). For estimand analyses, the EOT visit was aligned on the same nominal time scale from baseline using the windowing criteria in Supplementary Table 4. For a patient who already had an on-treatment assessment for the remapped nominal time window, the EOT visit was set to the subsequent visit.

Differences between arms were evaluated on four estimands (Supplementary Table 5). Estimand 1 evaluated between-group differences in the least squares (LS) mean changes from baseline to the end of induction (i.e., C4D1) and consolidation (i.e., C10D1) for each PRO domain. Data were analyzed using analysis of covariance (ANCOVA), with change from baseline to the timepoint of interest as the dependent variable and treatment arm, age randomization strata, and baseline PRO score as covariates. Estimand 2 assessed odds ratios (ORs) of meaningful improvement and deterioration at the end of induction and consolidation in each domain for ponatinib versus imatinib. A Cochran-Mantel-Haenszel model stratified by age randomization strata was used for OR estimations. Estimands 3 and 4 estimated HRs for time to confirmed improvement and deterioration, respectively, in each PRO domain for ponatinib versus imatinib. Confirmed improvement/deterioration was defined as meaningful improvement/deterioration (i.e., change from baseline ≥ the RD threshold prespecified before analysis) for ≥2 consecutive assessments [43, 44]. The Kaplan–Meier product-limit method was used to estimate the median time to confirmed improvement/deterioration. Between-group differences in time-to-event analyses were examined by stratified log-rank test and the HRs of ponatinib versus imatinib were estimated from stratified Cox proportional hazards regression. In both time-to-event (confirmed improvement and deterioration) analyses, patients with a missing baseline assessment or no post-baseline assessment were censored on the date of randomization. Patients who died before experiencing the event of interest or did not experience the event of interest were censored on the date of the last assessment with a non-missing PRO score. One exception for the analysis of time to confirmed improvement was that patients who prematurely discontinued treatment without first experiencing confirmed improvement were assumed to have no improvement for subsequent assessment visits and were censored on the date of the target nominal day (Supplementary Table 4) for the last assessment visit at which *n* was ≥10 for both treatment arms.

Missing data imputation was performed as a sensitivity analysis to assess the robustness of the results of estimand 1. Multiple imputation was conducted by using a control-based pattern mixture model [45] under the assumption of missing not at random. The purpose of the model was to use the observed values in the control (i.e., imatinib) group to derive the posterior distribution of the parameters from which the missing values in both control and treatment groups were imputed.

As the comparative assessment of treatment effects on PRO endpoints was an exploratory objective, no formal hypothesis testing or adjustment for multiplicity were performed [46]. Nevertheless, 95% CIs of the population-level summary and the corresponding nominal two-sided *p* values were estimated to help interpret treatment effects. A nominal *p* value below 0.05 was considered statistically significant. The Shapiro–Wilk test was performed to test the normality of the data, and Bartlett’s test was conducted to evaluate the homogeneity of variances. All statistical analyses were conducted using SAS® version 9.4 (SAS Institute, Cary, NC).

## Results

### Baseline patient characteristics and PRO scores

The ITT population comprised 245 patients (164 randomized to ponatinib and 81 to imatinib; Supplementary Fig. 1). Five patients in the ponatinib arm and two in the imatinib arm had no baseline or post-baseline PRO assessment and were excluded from the PRO-evaluable population. Death was the most common reason for study discontinuation, occurring in 21 (12.8%) patients in the ponatinib arm and 13 (16.0%) in the imatinib arm.

The proportion of patients not expected for PRO assessment was greater in the imatinib arm than in the ponatinib arm at all post-baseline visits, mostly due to treatment discontinuations related to receipt of HSCT or failure to achieve CR or MRD negativity (Supplementary Fig. 2). PRO completion rates were similar between the two arms and remained >70% up to cycle 19, after which the rates in the imatinib arm dropped and thereafter were lower than those in the ponatinib arm (Supplementary Table 6); however, the sample sizes were very small for assessment visits after cycle 19.

Patients in the PRO-evaluable population had a mean age of approximately 51 years in both arms, with 54.7% and 53.2% being female in the ponatinib and the imatinib arms, respectively. Baseline demographics and disease characteristics were generally well balanced between arms, except for a higher proportion of White patients in the imatinib arm (75.9% vs. 63.5% in the ponatinib arm; Table 1). Baseline PRO scores were generally comparable between arms (Supplementary Table 7). Patients had worse mean scores for FACT-G PWB, FACT-G EWB, FACT-G FWB, FACT-G total score, and EQ-VAS, compared with the reference populations [39, 40] (Supplementary Table 7).

**Table 1:** Baseline demographics and clinical characteristics (PRO-evaluable population).

Characteristic	Ponatinib (*n* = 159)	Imatinib (*n* = 79)
Age^a^ (years), mean (SD)	51.4 (16.0)	50.6 (14.8)
Age category, *n* (%)		
18 to <45 years	54 (34.0)	29 (36.7)
45 to <60 years	45 (28.3)	20 (25.3)
≥60 years	60 (37.7)	30 (38.0)
Sex, *n* (%)		
Female	87 (54.7)	42 (53.2)
Male	72 (45.3)	37 (46.8)
Race, *n* (%)		
American Indian or Alaska Native	2 (1.3)	2 (2.5)
Asian	20 (12.6)	11 (13.9)
Black or African American	8 (5.0)	4 (5.1)
White	101 (63.5)	60 (75.9)
Multiple	1 (0.6)	0
Not reported	27 (17.0)	2 (2.5)
Ethnicity, *n* (%)		
Hispanic or Latino	38 (23.9)	19 (24.1)
Not Hispanic or Latino	102 (64.2)	55 (69.6)
Unknown^b^	19 (11.9)	4 (6.3)
ECOG performance status, *n* (%)		
0	69 (43.4)	33 (41.8)
1	83 (52.2)	41 (51.9)
2	7 (4.4)	5 (6.3)
Time from initial diagnosis to first dose date of prior anticancer regimen (days), mean (SD)	15.2 (10.0)	15.9 (13.1)
Framingham score, mean (SD)	7.7 (10.6)	7.4 (8.5)
*BCR::ABL1* transcript type		
e1a2	110 (69.2)	51 (64.6)
e13/14a2	40 (25.2)	25 (31.6)
Undetermined/not tested/other	9 (5.7)	3 (3.8)
White blood cell count (10^9^/L), mean (SD)	12.9 (25.1)	8.1 (14.6)
Hemoglobin (g/L), mean (SD)	90.0 (16.7)	89.5 (16.6)
Platelets (10^9^/L), mean (SD)	80.3 (87.8)	85.4 (97.8)
Blast count (%), mean (SD)	68.1 (28.6)	64.0 (31.2)
Extramedullary disease, *n* (%)		
Yes	10 (6.3)	3 (3.8)
No	149 (93.7)	76 (96.2)

*ECOG* Eastern Cooperative Oncology Group, *PRO* patient-reported outcome, *SD* standard deviation.

^a^At informed consent.

^b^Including patients who did not report ethnicity and patients who reported their ethnicity as unknown.

### Change from baseline in PROs

**Fig. 1:**
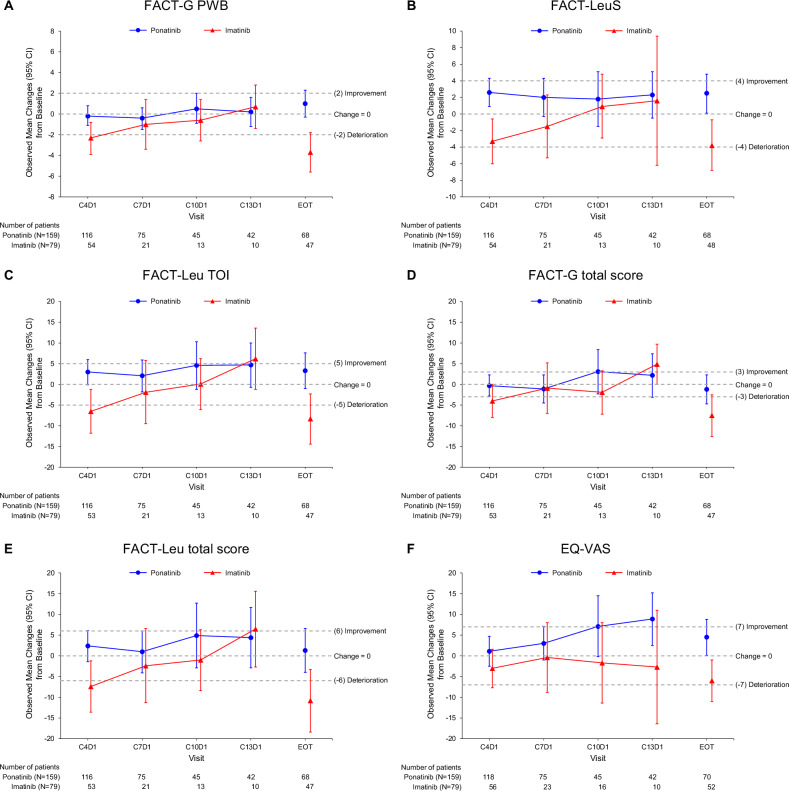
Observed mean changes from baseline for the primary domains of interest (PRO-evaluable population). **A** FACT-G PWB. **B** FACT-LeuS. **C** FACT-Leu TOI. **D** FACT-G total score. **E** FACT-Leu total score. **F** EQ-VAS. MID thresholds for improvement and worsening are shown as dotted lines. Data up to C13D1 (the latest visit at which *n* was ≥10 in both arms) and at EOT were included. C cycle, CI confidence interval, D day, EOT end of treatment, EQ-VAS EuroQol visual analogue scale, FACT-G Functional Assessment of Cancer Therapy–General, FACT-Leu Functional Assessment of Cancer Therapy–Leukemia, LeuS leukemia “additional concerns” subscale, MID minimal important difference, PRO patient-reported outcome, PWB physical well-being, TOI trial outcome index.

At the group level, observed mean changes from baseline showed that HRQoL in the primary domains of interest generally remained stable or improved over time in the ponatinib arm, with meaningful improvement in EQ-VAS at C10D1 and C13D1 (Fig. 1). The imatinib arm showed worsened HRQoL from baseline at most visits across the primary domains of interest, with meaningful deterioration observed at C4D1 and EOT for FACT-G PWB, FACT-Leu TOI, FACT-G total score, and FACT-Leu total score (Fig. 1). Meaningful improvement was seen with imatinib at C13D1 for FACT-Leu TOI, FACT-G total score, and FACT-Leu total score, but the sample size was small at that visit.

### Change from baseline in FACT-GP5 response

In both arms, most patients reported being “not at all” or “a little bit” bothered by treatment in response to FACT-GP5 across visits; the frequency was higher in the ponatinib arm than in the imatinib arm at all post-baseline visits (Supplementary Fig. 3). Additionally, the frequency of experiencing at least one level of worsening in FACT-GP5 from baseline were consistently higher with imatinib than with ponatinib across visits; the difference was greatest at EOT (Supplementary Fig. 4).

### Difference in least squares mean change from baseline in PROs (Estimand 1)

Differences in LS mean changes (95% CI) from baseline to the end of induction significantly (*p* < 0.05) favored ponatinib for four of the six primary domains of interest: FACT-G PWB (1.545 [0.204, 2.885]), FACT-LeuS (4.436 [2.077, 6.795]), FACT-Leu TOI (6.212 [1.670, 10.753]), and FACT-Leu total score (6.311 [0.645, 11.978]) (Table 2). For FACT-LeuS, FACT-Leu TOI, and FACT-Leu total score, the between-group differences were considered meaningful (i.e., exceeding the MID thresholds).

**Table 2:** Differences in LS mean changes from baseline for ponatinib versus imatinib (PRO-evaluable population).

Domain/subscale	Ponatinib		Imatinib		Difference in LS mean change (95% CI); *p*^†^	MID
	*n*	LS mean change (95% CI); p*	*n*	LS mean change (95% CI); p*		
Baseline to end of induction phase						
FACT-Leu						
**FACT-G PWB**	125	−0.303 (−1.076, 0.469); 0.440	63	−1.848 (−2.947, −0.749); 0.001	1.545 (0.204, 2.885); 0.024	2
FACT-G SWB	125	−1.435 (−2.134, −0.736); <0.001	63	−0.944 (−1.938, 0.050); 0.063	−0.491 (−1.704, 0.722); 0.425	2
FACT-G EWB	125	0.424 (−0.158, 1.006); 0.152	63	0.123 (−0.708, 0.955); 0.770	0.301 (−0.717, 1.318); 0.561	2
FACT-G FWB	125	0.083 (−0.830, 0.996); 0.858	62	−0.004 (−1.317, 1.309); 0.996	0.087 (−1.515, 1.689); 0.915	2
**FACT-LeuS**	125	2.296 (0.937, 3.655); 0.001	63	−2.140 (−4.073, −0.207); 0.030	4.436 (2.077, 6.795); <0.001	4
**FACT-Leu TOI**	125	2.102 (−0.495, 4.700); 0.112	62	−4.109 (−7.836, −0.383); 0.031	6.212 (1.670, 10.753); 0.008	5
**FACT-G TS**	125	−1.156 (−3.361, 1.049); 0.302	62	−2.877 (−6.041, 0.287); 0.074	1.721 (−2.134, 5.576); 0.380	3
**FACT-Leu TS**	125	1.172 (−2.070, 4.414); 0.477	62	−5.139 (−9.790, −0.488); 0.031	6.311 (0.645, 11.978); 0.029	6
EQ-5D-5L						
UK-based HUI	127	−0.034 (−0.067, −0.001); 0.042	66	−0.040 (−0.086, 0.007); 0.093	0.005 (−0.051, 0.062); 0.850	0.08
US-based HUI	127	−0.024 (−0.048, 0.000); 0.053	66	−0.027 (−0.061, 0.007); 0.119	0.003 (−0.038, 0.044); 0.884	0.08
**EQ-VAS**	127	0.952 (−1.953, 3.857); 0.519	66	−1.892 (−5.968, 2.183); 0.361	2.845 (−2.144, 7.834); 0.262	7
Baseline to end of consolidation phase						
FACT-Leu						
**FACT-G PWB**	101	0.437 (−0.481, 1.355); 0.349	53	−2.506 (−3.778, −1.234); <0.001	2.943 (1.373, 4.513); <0.001	2
FACT-G SWB	101	−1.226 (−2.074, −0.378); 0.005	54	−1.312 (−2.474, −0.149); 0.027	0.086 (−1.350, 1.521); 0.906	2
FACT-G EWB	101	0.232 (−0.531, 0.996); 0.549	54	−0.843 (−1.895, 0.209); 0.115	1.075 (−0.228, 2.378); 0.105	2
FACT-G FWB	101	0.224 (−0.852, 1.300); 0.681	54	−0.974 (−2.451, 0.503); 0.194	1.199 (−0.627, 3.025); 0.197	2
**FACT-LeuS**	101	1.465 (−0.177, 3.106); 0.080	54	−1.657 (−3.913, 0.598); 0.149	3.122 (0.327, 5.918); 0.029	4
**FACT-Leu TOI**	101	2.267 (−0.812, 5.347); 0.148	53	−4.981 (−9.249, −0.713); 0.022	7.248 (1.977, 12.520); 0.007	5
**FACT-G TS**	101	−0.298 (−2.946, 2.349); 0.824	53	−5.524 (−9.189, −1.860); 0.003	5.226 (0.708, 9.744); 0.024	3
**FACT-Leu TS**	101	1.266 (−2.665, 5.197); 0.526	53	−6.998 (−12.443, −1.552); 0.012	8.264 (1.543, 14.984); 0.016	6
EQ-5D-5L						
UK-based HUI	103	−0.006 (−0.046, 0.034); 0.775	59	−0.051 (−0.104, 0.002); 0.058	0.045 (−0.021, 0.111); 0.178	0.08
US-based HUI	103	0.002 (−0.028, 0.032); 0.908	59	−0.035 (−0.075, 0.005); 0.086	0.037 (−0.013, 0.087); 0.150	0.08
**EQ-VAS**	103	5.298 (1.848, 8.748); 0.003	59	−2.490 (−7.067, 2.087); 0.284	7.788 (2.054, 13.523); 0.008	7

Domains/subscales in bold are primary domains of interest. Values exceeding the MID threshold are underlined.

*CI* confidence interval, *EQ-VAS* EuroQol visual analogue scale, *EWB* emotional well-being, *FACT-G* Functional Assessment of Cancer Therapy–General, *FACT-Leu* Functional Assessment of Cancer Therapy–Leukemia, *FWB* functional well-being, *HUI* health utility index, *LeuS* leukemia “additional concerns” subscale, *LS* least squares, *MID* minimal important difference, *PRO* patient-reported outcome, *PWB* physical well-being, *SWB* social/family well-being, *TOI* trial outcome index, *TS* total score.

*Nominal *p* for within-group LS mean change from baseline.

^†^Nominal *p* for between-group difference in LS mean change from baseline.

Differences in the LS mean changes from baseline to the end of consolidation significantly favored ponatinib for all primary domains of interest: FACT-G PWB (2.943 [1.373, 4.513]), FACT-LeuS (3.122 [0.327, 5.918]), FACT-Leu TOI (7.248 [1.977, 12.520]), FACT-G total score (5.226 [0.708, 9.744]), FACT-Leu total score (8.264 [1.543, 14.984]), EQ-VAS (7.788 [2.054, 13.523]) (Table 2). For all these domains except for FACT-LeuS, the differences were also meaningful.

Results of the sensitivity analysis with missing data imputation were generally consistent with those from the main analyses (Supplementary Table 8). However, as the control-based missing data imputation is a conservative approach, the differences, as expected, were of a smaller magnitude and lost statistical significance for FACT-G PWB and FACT-Leu total score (baseline to end of induction), as well as for FACT-G total score, FACT-Leu total score, and EQ-VAS (baseline to end of consolidation), compared with the main analysis results.

### Difference in proportions of patients with meaningful improvement (and deterioration) (Estimand 2)

**Fig. 2:**
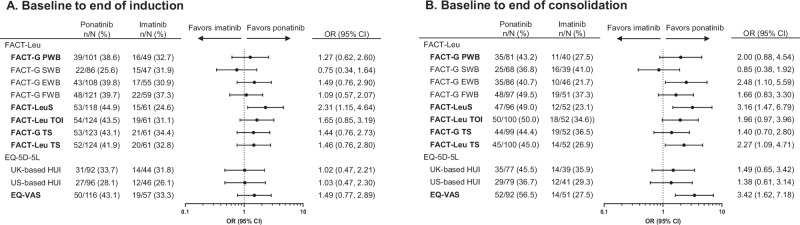
Difference in proportions of patients experiencing meaningful improvement from baseline (PRO-evaluable population). **A** Meaningful improvement from baseline to end of induction. **B** Meaningful improvement from baseline to end of consolidation. Domains/subscales in bold are primary domains of interest. “*N*” indicates the number of patients in the PRO-evaluable population who were at risk of the event of interest (meaningful improvement) and had a non-missing score change at the time point of interest, and “*n*” indicates the number of patients with meaningful improvement. CI confidence interval, EQ-VAS EuroQol visual analogue scale, EWB emotional well-being, FACT-G Functional Assessment of Cancer Therapy–General, FACT-Leu Functional Assessment of Cancer Therapy–Leukemia, FWB functional well-being, HUI health utility index, LeuS leukemia “additional concerns” subscale, OR odds ratio, PRO patient-reported outcome, PWB physical well-being, SWB social/family well-being, TOI trial outcome index, TS total score.

**Fig. 3:**
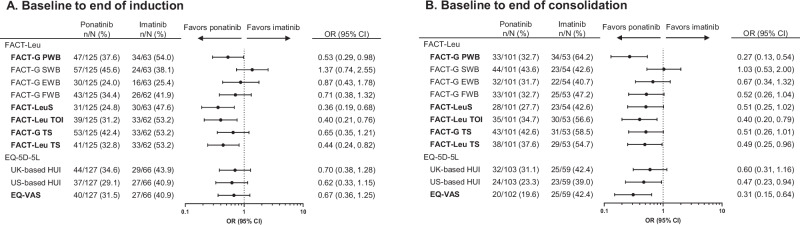
Difference in proportions of patients experiencing meaningful deterioration from baseline (PRO-evaluable population). **A** Meaningful deterioration from baseline to end of induction. **B** Meaningful deterioration from baseline to end of consolidation. Domains/subscales in bold are primary domains of interest. “*N*” indicates the number of patients in the PRO-evaluable population who were at risk of the event of interest (meaningful deterioration) and had a non-missing score change at the time point of interest, and “*n*” indicates the number of patients with meaningful deterioration. CI confidence interval, EQ-VAS EuroQol visual analogue scale, EWB emotional well-being, FACT-G Functional Assessment of Cancer Therapy–General, FACT-Leu Functional Assessment of Cancer Therapy–Leukemia, FWB functional well-being, HUI health utility index, LeuS leukemia “additional concerns” subscale, OR odds ratio, PRO patient-reported outcome, PWB physical well-being, SWB social/family well-being, TOI trial outcome index, TS total score.

Patients in the ponatinib arm were significantly more likely to experience meaningful improvement in FACT-LeuS at the end of induction and in FACT-G EWB, FACT-LeuS, FACT-Leu total score, and EQ-VAS at the end of consolidation than those in the imatinib arm (Fig. 2A, B). Similarly, patients treated with ponatinib were significantly less likely than those treated with imatinib to experience meaningful deterioration in FACT-G PWB, FACT-LeuS, FACT-leu TOI, and FACT-Leu total score at the end of induction and in FACT-G PWB, FACT-Leu TOI, FACT-Leu total score, EQ-VAS, and EQ-5D-5L HUI (US-based) at the end of consolidation (Fig. 3A, B).

### Time to confirmed improvement and deterioration (Estimands 3 and 4)

The ponatinib arm had numerically higher cumulative probability of experiencing a confirmed improvement by week 36 (end of consolidation) (Supplementary Table 9) and shorter median times to confirmed improvement than the imatinib arm across all 6 primary domains of interest. The HRs (95% CI) significantly favored ponatinib for FACT-LeuS (2.24 [1.12, 4.48]), FACT-Leu TOI (2.01 [1.03, 3.90]), and EQ-VAS (3.33 [1.57, 7.07]).

The cumulative probability of experiencing a confirmed deterioration by week 36 in the ponatinib arm was numerically lower or similar to that in the imatinib arm for all primary domains of interest (Supplementary Table 9). Median time to confirmed deterioration was not estimable for most domains of interest in both arms. The risk of experiencing a confirmed deterioration in EQ-VAS was significantly lower with ponatinib than imatinib (HR 0.51; 95% CI: 0.27, 0.96). For other domains of interest, no significant difference was found between the two arms.

## Discussion

Results from the current post hoc PRO analysis from PhALLCON suggest a more favorable effect of ponatinib than imatinib in patients with newly diagnosed Ph+ ALL, in line with the efficacy outcomes from the trial [13]. At baseline, PhALLCON patients had worse HRQoL than the reference populations [39–41], which is not unexpected for patients with ALL [47]. Observed changes from baseline showed that patients treated with ponatinib generally reported a trend of stable or improving PRO scores over time for the primary domains of interest. In contrast, patients treated with imatinib showed a more mixed trend, with meaningful deterioration at C4D1 (i.e., end of induction) for several primary domains, followed by gradual improvement over time. This may be attributable to patients with improvement being more likely to continue treatment. Comparative assessments showed that ponatinib improved or maintained HRQoL compared with imatinib across primary domains of interest, regardless of the estimand used. Overall treatment tolerability, as assessed by the FACT-GP5, was generally more favorable with ponatinib than with imatinib. Moreover, the robustness of the main analysis findings for the estimand 1 was supported by those from the sensitivity analyses using a conservative control-based multiple imputation approach to address the issue of missing data.

PROs are increasingly incorporated as secondary or exploratory endpoints in blood cancer trials to capture patient experience [48]. For example, PRO data from the phase 3 INO-VATE trial demonstrated better HRQoL in patients with relapsed/refractory ALL who received inotuzumab ozogamicin (InO) compared with those receiving standard-of-care chemotherapies [24]. Similarly, PRO data from the phase 3 Viale-C trial suggested longer HRQoL maintenance with venetoclax plus low-dose cytarabine (LDAC) compared with LDAC alone in patients with acute myeloid leukemia ineligible for intensive chemotherapy [23]. The PRO findings from these trials provide valuable insights into patient experience and complement clinical efficacy data, as improved HRQoL was observed alongside significantly higher CR rates with InO and venetoclax plus LDAC. While overall survival differences were not statistically significant [49, 50], the PRO results underscore the importance of evaluating patient experience alongside traditional endpoints. These findings support the routine inclusion of PROs in acute leukemia trials for a more comprehensive assessment of treatment impact.

The results from the present PRO analysis from PhALLCON add to the body of evidence supporting the superior treatment effect of ponatinib over imatinib in Ph+ ALL by showing a more favorable effect of ponatinib on how patients feel and function. Our findings underscore the significance of incorporating PROs in clinical trials of ALL treatments, as PRO data can provide timely support for regulatory decisions regarding the potential benefits of the treatment [17]. This becomes especially relevant when survival data are unlikely to be mature at the time of regulatory review and a surrogate endpoint (e.g., the MRD-negative CR rate in PhALLCON) is used as the primary endpoint, since the treatment effect on overall survival may not always correlate well with that of a surrogate [15, 17].

Furthermore, while rates of treatment-emergent adverse events, as conventionally reported by clinicians or study investigators, were similar between the treatment arms in PhALLCON [13], FACT-GP5 responses tended to show that patients in the ponatinib arm felt less bothered by the side effects of study treatment than those in the imatinib arm. This highlights the importance of directly capturing the patient’s voice and experience with treatment when assessing tolerability.

To our knowledge, this is the first report comparing the effects of ponatinib and imatinib on PROs or HRQoL in patients with Ph+ ALL. The application of the ICH E9(R1) estimand framework [42] in the comparative assessment of PRO endpoints in this study is a notable methodological strength. The estimand framework provides a structured approach to defining, analyzing, and interpreting treatment effects, making the results more transparent and reliable. This could set a precedent for future studies, demonstrating the feasibility and value of applying the estimand framework to PRO analysis and reporting for oncology trials.

The interpretation of the treatment effect on HRQoL should be based on the context defined by the attributes of an estimand, particularly the specific strategies employed to address selected ICEs. In our analysis, the treatment policy strategy, which assesses treatment effects regardless of the occurrence of ICEs, was selected to handle the impact of premature treatment discontinuations for any cause other than death because it reflects real-world practice and is close to the traditional ITT principle. As death is a terminal event and cannot be handled using the treatment policy strategy, the while-on-treatment strategy (i.e., assessing treatment effect before the occurrence of the ICE) and hypothetical strategy (i.e., assessing treatment effect in the absence of the ICE) were used to handle the impact of death in different estimands. All these strategies led to consistent findings in favor of ponatinib. Additionally, the frequency of death in the study were small and similar between arms, as of the current data cutoff. Therefore, death as an ICE was unlikely to have had any major impact on the results, regardless of the ICE strategy used.

The study has limitations. First, the PRO endpoints were evaluated as an exploratory objective in PhALLCON, and no adjustment was performed for multiple testing, therefore the nominally significant results should be interpreted with caution. Second, the current analyses could have been impacted by missing data, resulting in reduced statistical power and biased estimations. However, the findings from the sensitivity analysis with a conservative multiple imputation approach support the robustness of the results. Third, the analysis of LS mean changes from baseline focuses on outcomes at the end of induction and consolidation. While these are important milestones in treatment, they may not fully capture the long-term treatment impact on quality of life and functioning, which are crucial aspects of patient experience. Additionally, selection bias is possible in the current study, particularly for PRO data collected after the end of induction. Per the study design, patients who did not achieve MRD-negative CR at the end of induction discontinued study treatment, with a few exceptions if the patient was MRD-negative with incomplete hematologic remission, at the investigator’s discretion. Thus, PRO data from the later visits tended to reflect the HRQoL of patients with response to and/or good tolerance of treatment. Among patients who discontinued study treatment, PRO scores were worse in those stopping imatinib, as evidenced by the worse mean score changes from baseline to EOT for most primary domains.

In conclusion, PRO data from the phase 3 PhALLCON trial suggest improvements in quality of life/functioning and leukemia-specific symptoms with ponatinib relative to imatinib, when combined with reduced-intensity chemotherapy, in patients with newly diagnosed Ph+ ALL. Results from descriptive analyses of FACT-GP5 response suggest a more favorable overall treatment tolerability with ponatinib than imatinib. Our results bolster the clinical efficacy and safety findings from PhALLCON and provide further evidence supporting the use of ponatinib, in combination with reduced-intensity chemotherapy, as a frontline treatment for Ph+ ALL.

## Supplementary information

Supplemental material

## Data Availability

The data sets, including the redacted study protocol, redacted statistical analysis plan, and individual participant data of the completed study supporting the results reported in this article, will be made available within 3 months from initial request to researchers who provide a methodologically sound proposal. The data will be provided after de-identification, in compliance with applicable privacy laws, data protection, and requirements for consent and anonymization.

## Code availability

The computer codes used to generate results reported in this paper may be available upon request.
