# Supplementary material for: Patient-reported outcomes in Philadelphia chromosome-positive acute lymphoblastic leukemia patients treated with ponatinib or imatinib: results from the PhALLCON trial
Source: Leukemia. 2025 Apr 16;39(6):1342–50. doi: 10.1038/s41375-025-02608-4 (PMC12133573; doi:10.1038/s41375-025-02608-4)
Supplement: Supplementary file 1 — Supplemental material [file 41375_2025_2608_MOESM1_ESM.pdf]

Patient-reported outcomes in Ph+ ALL patients treated with ponatinib or imatinib: results from the PhALLCON trial

Ajibade Ashaye, Ling Shi, Ibrahim Aldoss, Pau Montesinos, Pankit Vachhani, Vanderson Rocha, Cristina Papayannidis, Jessica T. Leonard, Maria R. Baer, Jose-Maria Ribera, James McCloskey, Jianxiang Wang, Sujun Gao, Deepali Rane, Shien Guo

Supplemental Data

Table of Contents

Supplemental Table 1. PRO assessment schedule 2

Supplemental Table 2. FACT-Leu score ranges 3

Supplemental Table 3. Meaningful improvement and deterioration thresholds for FACT-Leu and EQ-5D-5L domains. 4

Supplemental Table 4. Time windows for PRO assessments (for remapping EOT visit only).. 5

Supplemental Table 5. Main attributes of estimands 6

Supplemental Table 6. Completion rates for the FACT-Leu and EQ-5D-5L in the ITT population. 8

Supplemental Table 7. Baseline PRO scores in the PRO-evaluable population 9

Supplemental Table 8. Sensitivity analysis of differences in LS mean changes from baseline for the primary domains of interest 10

Supplemental Table 9. Time to confirmed improvement and deterioration (PRO-evaluable population) 11

Supplemental Figure 1. Patient disposition. 13

Supplemental Figure 2. Proportions of patients in the ITT population eligible and ineligible for PRO assessments over time 14

Supplemental Figure 3. Distribution of responses for FACT-GP5 (PRO-evaluable population). 15

Supplemental Figure 4. Distribution of changes from baseline in response level for FACT-GP5 (PRO-evaluable population) 16

Appendix. PhALLCON Institutional Review Board/Independent Ethics Committees 17

Supplemental Table 1. PRO assessment schedule

	Screening/ baseline	Induction (C1 to C3)	Consolidation (C4 to C9)		Maintenance (C10 to C20)				Single-agent Tx*	EOT†
		C1D1	C4D1	C7D1	C10D1	C13D1	C16D1	C19D1	C21D1 and Q6C	
FACT-Leu	x	x	x	x	x	x	x	x	x	x
EQ-5D-5L	x	x	x	x	x	x	x	x	x	x

Baseline was defined as the last observed value before the first dose of study medication (i.e., C1D1 or as the screening visit if the C1D1 assessment was missing).
C, cycle; D, day; EOT, end of treatment; FACT-Leu, Functional Assessment of Cancer Therapy–Leukemia; HSCT, hematopoietic stem cell transplantation; PRO, patient-reported outcome; Q6C, every 6 cycles; Tx, treatment.
*****After the end of Cycle 20, patients received single-agent ponatinib or imatinib until they experienced relapse from complete remission progressive disease, had unacceptable toxicity, withdrew consent, proceeded to HSCT or alternative therapy, or completed the study, whichever occurred first.
†The EOT visit was expected to occur 30 + 7 days after a patient had discontinued the study drug (day of last dose) but could occur <30 days after discontinuation of study drug because of the requirement that the EOT visit occur before HSCT or before initiation of any alternative treatment.

Supplemental Table 2. FACT-Leu score ranges

Domain/subscale	Domains summarized	Number of items	Score range
FACT-G PWB	NA	7	0–28
FACT-G SWB	NA	7	0–28
FACT-G EWB	NA	6	0–24
FACT-G FWB	NA	7	0–28
FACT-LeuS	NA	17	0–68
FACT-Leu TOI	PWB + FWB + FACT-LeuS	31	0–124
FACT-G total score	PWB + SWB + EWB +FWB	27	0–108
FACT-Leu total score	FACT-G + FACT-LeuS	44	0–124

For each item, responses range from 0 (“not at all”) to 4 (“very much”). Items for which a higher value indicates worse HRQoL were reverse scored so that a higher score indicated better HRQoL. Domain scores were calculated only if >50% of the items were non-missing. FACT-G total score, FACT-Leu total score, and FACT-Leu TOI were calculated only if >80% of all items were non-missing.

EWB, emotional well-being; FACT-G, Functional Assessment of Cancer Therapy – General; FACT-Leu, Functional Assessment of Cancer Therapy – Leukemia; FWB, functional well-being; HRQoL, health-related quality of life; LeuS, leukemia “additional concerns” subscale; PWB, physical well-being; SWB, social/family well-being; TOI, trial outcome index.

Supplemental Table 3. Meaningful improvement and deterioration thresholds for FACT-Leu and EQ-5D-5L domains.

Domain/subscale	MID and RD thresholds	
	**Improvement**	**Deterioration**
FACT-Leu		
FACT-G PWB	+2	−2
FACT-G SWB	+2	−2
FACT-G EWB	+2	−2
FACT-G FWB	+2	−2
FACT-LeuS	+4	−4
FACT-G total score	+3	−3
FACT-Leu total score	+6	−6
FACT-Leu TOI	+5	−5
EQ-5D-5L		
HUI (UK and US values)	+0.08	−0.08
EQ-VAS	+7	−7

MID/RD thresholds are based on published values.[26-31]

EQ-VAS, EuroQol visual analogue scale; EWB, emotional well-being; FACT-G, Functional Assessment of Cancer Therapy – General; FACT-Leu, Functional Assessment of Cancer Therapy – Leukemia; FWB, functional well-being; HUI, health utility index; LeuS, leukemia "additional concerns" subscale; MID, minimal important difference; PWB, physical well-being; RD, responder definition; SWB, social/family well-being; TOI, trial outcome index.

Supplemental Table 4. Time windows for PRO assessments (for remapping EOT visit only)

Nominal timepoint	Target nominal day (relative to the treatment start date)	Time window for PRO assessment (relative to the treatment start date)
C1D1 (Week 1)	1	0–28 (target nominal day +27/−1 days)
C4D1 (Week 13)	85	29–112 (target nominal day +27/−56 days)
C7D1 (Week 25)	169	113–196 (target nominal day +27/−56 days)
C10D1 (Week 37)	253	197–280 (target nominal day +27/−56 days)
C13D1 (Week 49)	337	281–364 (target nominal day +27/−56 days)
C16D1 (Week 61)	421	365–448 (target nominal day +27/−56 days)
C19D1 (Week 73)	505	449–532 (target nominal day +27/−56 days)
C21D1 (Week 81)	561	533–645 (target nominal day +84/−28 days)
C27D1 (Week 105)	729	646–813 (target nominal day +84/−83 days)
C33D1 (Week 129)	897	814–981 (target nominal day +84/−83 days)
….		
CXD1	(X-1)*28 +1	Target nominal day +84/−83

For EOT data to be utilized in the estimand analyses, the EOT visits were aligned on the same nominal time scale from baseline based on 28 days/cycle. If a participant already had an on-treatment assessment for the remapped nominal time window, then the EOT visit was set to the subsequent visit.
C, cycle; D, day; EOT, end of treatment; PRO, patient-reported outcome.

Supplemental Table 5. Main attributes of estimands

**Research question and attributes**	**Estimand 1**	**Estimand 2**	**Estimand 3**	**Estimand 4**
Research question	Was ponatinib superior to imatinib on a given PRO domain up to the the end of induction (and consolidation) or death, whichever occurred first, regardless of premature treatment discontinuation due to any other cause?	Was ponatinib superior to imatinib on the proportion of patients experiencing meaningful improvement (and deterioration) in a given PRO domain from baseline to the end of induction (and consolidation) or death, whichever occurred first, regardless of premature treatment discontinuation for any other cause?	Was ponatinib superior to imatinib on time to improvement in a given PRO domain, in the absence of death and regardless of premature treatment discontinuation due to any other cause?	Was ponatinib superior to imatinib in time to deterioration in a given PRO domain, in the absence of death and regardless of premature treatment discontinuation due to any other cause?
Treatment	Ponatinib vs imatinib	Ponatinib vs imatinib	Ponatinib vs imatinib	Ponatinib vs imatinib
Population	PRO-evaluable population	PRO-evaluable population, excluding patients whose baseline scores precluded them from achieving meaningful improvement or deterioration	PRO-evaluable population, excluding patients whose baseline scores precluded them from achieving meaningful improvement	PRO-evaluable population, excluding patients whose baseline scores precluded them from achieving meaningful deterioration
Endpoint	Change from baseline to the end of induction and consolidation	Proportions of patients experiencing clinically meaningful improvement or deterioration from baseline to the end of induction and consolidation	Time to confirmed improvement	Time to confirmed deterioration

Handling of ICEs	Death: while-on-treatment strategy<br><br>Premature treatment discontinuation due to any other cause*: treatment policy strategy	Death: while-on-treatment strategy<br><br>Premature treatment discontinuation due to any other cause*: treatment policy strategy	Death: hypothetical strategy<br><br>Premature treatment discontinuation due to any other cause*: treatment policy strategy	Death: hypothetical strategy<br><br>Premature treatment discontinuation due to any other cause*: treatment policy strategy
Population-level summary	Between-group difference (95% CI, nominal *P* value) in LS mean changes at the end of induction and consolidation	Between-group OR (95% CI, nominal *P* value) for the proportion of patients experiencing meaningful improvement (and deterioration) from baseline during induction and consolidation	Stratified log-rank test for treatment comparison and HR (95% CI, nominal *P* value) for effect size	Stratified log-rank test for treatment comparison and HR (95% CI, nominal *P* value) for effect size

Hypothetical strategy: death was assumed to have not occurred.
Treatment policy strategy: for premature treatment discontinuations due to any other cause, data captured post the event, including those captured at the EOT visit, were considered in the analysis.
While-on-treatment strategy: for deaths occurred before the time point of interest, the last non-missing PRO assessment prior to the death was considered in the analyses.
CI, confidence interval; EOT, end of treatment; HSCT, hematopoietic stem cell transplantation; HR, hazard ratio; ICE, intercurrent event; LS, least squares; MRD, minimal residual disease; OR, odds ratio; PRO, patient-reported outcome.
*Cause other than death, such as failure to achieve MRD-negative complete remission, relapse from complete remission or progressive disease, receipt of HSCT or alternative therapy, or unacceptable treatment toxicity

Supplemental Table 6. Completion rates for the FACT-Leu and EQ-5D-5L in the ITT population.

	FACT-Leu, n/N (%)		EQ-5D-5L, n/N (%)	
	**Ponatinib**	**Imatinib**	**Ponatinib**	**Imatinib**
Baseline	150/164 (91.5)	74/81 (91.4)	154/164 (93.9)	76/81 (93.8)
C4D1	122/147 (83.0)	55/63 (87.3)	123/147 (83.7)	57/63 (90.5)
C7D1	77/89 (86.5)	23/27 (85.2)	77/89 (86.5)	23/27 (85.2)
C10D1	46/59 (78.0)	15/18 (83.3)	46/59 (78.0)	16/18 (88.9)
C13D1	43/54 (79.6)	10/12 (83.3)	43/54 (79.6)	10/12 (83.3)
C16D1	36/45 (80.0)	7/10 (70.0)	36/45 (80.0)	8/10 (80.0)
C19D1	34/42 (81.0)	7/9 (77.8)	34/42 (81.0)	7/9 (77.8)
C21D1	28/36 (77.8)	3/8 (37.5)	29/36 (80.6)	3/8 (37.5)
C27D1	14/20 (70.0)	1/6 (16.7)	16/20 (80.0)	2/6 (33.3)
C33D1	6/8 (75.0)	1/3 (33.3)	8/9 (88.9)	1/3 (33.3)
C39D1	1/1 (100.0)	1/2 (50.0)	1/1 (100.0)	1/2 (50.0)
EOT	69/85 (81.2)	52/65 (80.0)	70/85 (82.4)	54/65 (83.1)

"N" indicates the number of ITT patients who were still expected to provide PRO assessment at a given visit, and "n" indicates the number of patients who completed PRO assessment at that visit.
C, cycle; D, day; EOT, end of treatment. FACT-Leu, Functional Assessment of Cancer Therapy – Leukemia; ITT, intent-to-treat.

Supplemental Table 7. Baseline PRO scores in the PRO-evaluable population

Domain/subscale	Mean (SD)		Reference population norm*, mean
	Ponatinib	Imatinib	
FACT-Leu	n = 150	n = 74	
**FACT-G PWB**	21.9 (5.0)	22.5 (4.6)	24.8
FACT-G SWB	22.9 (4.6)	22.1 (5.3)	19.4
FACT-G EWB	17.3 (4.4)	18.3 (4.2)	21.2
FACT-G FWB	14.9 (6.0)	16.1 (6.3)	20.0
**FACT-LeuS**	49.1 (10.2)	50.2 (9.2)	-
**FACT-Leu TOI**	85.8 (18.9)	88.8 (17.3)	-
**FACT-G total score**	77.0 (16.3)	79.0 (15.7)	85.5
**FACT-Leu total score**	126.1 (24.8)	129.2 (22.9)	-
EQ-5D-5L	n = 154	n = 76	
UK-based HUI	0.79 (0.20)	0.81 (0.18)	0.84
US-based HUI	0.83 (0.15)	0.84 (0.14)	0.84
**EQ-VAS**	71.2 (19.7)	72.7 (18.3)	82.5

Domains/subscales in bold are primary domains of interest.
EQ-VAS, EuroQol visual analogue scale; EWB, emotional well-being; FACT-G, Functional Assessment of Cancer Therapy – General; FACT-Leu, Functional Assessment of Cancer Therapy – Leukemia; FWB, functional well-being; HUI, health utility index; LeuS, leukemia “additional concerns” subscale; PRO, patient-reported outcome; PWB, physical well-being; SD, standard deviation; SWB, social/family well-being; TOI, trial outcome index.
*FACT-Leu norm scores were based on the Austrian general population (N=926).[32] HUI and EQ-VAS norm scores were based on the UK general population.[33] All norm scores were reweighted by the age-by-gender distributions of the PRO-evaluable population of this study.

Supplemental Table 8. Sensitivity analysis of differences in LS mean changes from baseline for the primary domains of interest

Domain/subscale	Ponatinib (n = 159) LS mean change (95% CI); *P**	Imatinib (n = 79) LS mean change (95% CI); *P**	Difference in LS mean change (95% CI); *P*†	MID
**Baseline to end of induction phase**				
FACT-G PWB	−0.732 (−1.616, 0.152); 0.104	−2.063 (−3.322, −0.805); 0.001	1.331 (−0.212, 2.874); 0.090	2
FACT-LeuS	1.346 (−0.298, 2.991); 0.108	−2.410 (−4.601, −0.219); 0.031	3.757 (0.984, 6.529); 0.008	4
FACT-Leu TOI	1.310 (−1.896, 4.515); 0.421	−4.510 (−8.472, −0.547); 0.026	<u>5.819</u> (0.915, 10.723); 0.020	5
FACT-G TS	−1.789 (−4.151, 0.573); 0.137	−3.230 (−6.249, −0.211); 0.036	1.441 (−2.345, 5.226); 0.455	3
FACT-Leu TS	−0.154 (−3.882, 3.574); 0.935	−5.563 (−10.289, −0.838); 0.021	5.409 (−0.469, 11.288); 0.071	6
EQ-VAS	0.969 (−1.902, 3.840); 0.508	−1.994 (−6.257, 2.268); 0.358	2.963 (−2.093, 8.019); 0.250	7
**Baseline to end of consolidation phase**				
FACT-G PWB	0.138 (−0.826, 1.101); 0.778	−2.007 (−3.224, −0.789); 0.001	<u>2.144</u> (0.738, 3.550); 0.003	2
FACT-LeuS	1.380 (−0.170, 2.929); 0.081	−1.234 (−3.244, 0.777); 0.229	2.613 (0.219, 5.008); 0.032	4
FACT-Leu TOI	1.874 (−1.363, 5.111); 0.255	−3.895 (−8.107, 0.318); 0.070	<u>5.769</u> (1.018, 10.519); 0.017	5
FACT-G TS	−1.559 (−5.077, 1.960); 0.380	−4.501 (−8.696, −0.305); 0.036	2.942 (−1.961, 7.845); 0.238	3
FACT-Leu TS	−0.115 (−4.552, 4.323); 0.959	−5.938 (−11.437, −0.439); 0.034	5.823 (−0.620, 12.266); 0.076	6
EQ-VAS	4.229 (1.054, 7.405); 0.009	−1.337 (−5.531, 2.857); 0.532	5.566 (0.397, 10.735); 0.035	7

Values exceeding the MID threshold are underlined.
CI, confidence interval; EQ-VAS, EuroQol visual analogue scale; FACT-G, Functional Assessment of Cancer Therapy – General; FACT-Leu, Functional Assessment of Cancer Therapy – Leukemia; LeuS, leukemia "additional concerns" subscale; LS, least squares; MID, minimal important difference; PWB, physical well-being; TOI, trial outcome index; TS, total score.
*Nominal *P* for within-group LS mean change from baseline.
†Nominal *P* for between-group difference in LS mean change from baseline.

Supplemental Table 9. Time to confirmed improvement and deterioration (PRO-evaluable population)

Domain/subscale	Ponatinib			Imatinib			HR (95% CI)†	Nominal *P*
	n*	Cumulative % by week 36 (95% CI)	Median time to confirmed improvement or deterioration (95% CI), weeks	n*	Cumulative % by week 36 (95% CI)	Median time to confirmed improvement or deterioration (95% CI), weeks		
**Time to confirmed improvement**								
FACT-Leu								
**FACT-G PWB**	131	26.5 (18.3, 35.4)	104.0 (61.0, NE)	62	12.4 (5.0, 23.4)	NE (NE, NE)	2.17 (0.95, 4.95)	0.065
FACT-G SWB	112	18.4 (11.0, 27.3)	104.0 (72.0, NE)	60	25.8 (14.2, 39.1)	66.1 (66.1, NE)	0.73 (0.37, 1.45)	0.369
FACT-G EWB	139	23.8 (16.2, 32.2)	76.3 (73.7, NE)	71	12.2 (5.3, 22.1)	NE (58.9, NE)	1.83 (0.87, 3.86)	0.111
FACT-G FWB	155	28.2 (20.5, 36.3)	73.6 (60.6, NE)	76	25.0 (14.8, 36.5)	49.1 (49.1, NE)	1.16 (0.65, 2.07)	0.619
**FACT-LeuS**	151	31.9 (23.6, 40.4)	100.9 (61.6, NE)	77	12.8 (5.9, 22.4)	NE (NE, NE)	2.24‡ (1.12, 4.48)	0.023
**FACT-Leu TOI**	158	29.1 (21.4, 37.2)	73.7 (62.4, NE)	78	14.4 (7.0, 24.3)	NE (NE, NE)	2.01‡ (1.03, 3.90)	0.040
**FACT-G TS**	157	30.4 (22.5, 38.7)	76.3 (72.0, NE)	78	22.8 (13.2, 34.0)	NE (NE, NE)	1.31 (0.72, 2.37)	0.374
**FACT-Leu TS**	158	29.2 (21.5, 37.3)	74.7 (61.4, NE)	78	17.7 (9.4, 28.2)	NE (56.0, NE)	1.61 (0.87, 3.01)	0.132
EQ-5D-5L								
UK-based HUI	117	25.7 (17.3, 35.0)	NE (56.9, NE)	55	17.3 (8.0, 29.5)	NE (71.1, NE)	1.43 (0.67, 3.06)	0.349
US-based HUI	121	20.2 (12.9, 28.6)	NE (61.3, NE)	57	14.5 (6.3, 25.9)	NE (71.1, NE)	1.36 (0.61, 3.04)	0.451
**EQ-VAS**	141	33.7 (25.2, 42.5)	82.6 (50.9, NE)	70	8.4 (3.0, 17.2)	NE (49.1, NE)	3.33‡ (1.57, 7.07)	0.002
**Time to confirmed deterioration**								
FACT-Leu								
**FACT-G PWB**	159	25.0 (17.1, 33.7)	NE (61.1, NE)	79	32.6 (20.4, 45.4)	72.1 (44.0, NE)	0.61 (0.35, 1.08)	0.089
FACT-G SWB	159	29.6 (21.3, 38.4)	NE (48.0, NE)	79	27.0 (14.3, 41.3)	NE (40.9, NE)	1.03 (0.57, 1.88)	0.922
FACT-G EWB	159	19.0 (11.6, 27.8)	NE (NE, NE)	79	21.0 (8.7, 37.0)	NE (40.1, NE)	0.73 (0.34, 1.57)	0.428

Domain/subscale	Ponatinib			Imatinib			HR (95% CI)†	Nominal *P*
	**n***	**Cumulative % by week 36 (95% CI)**	**Median time to confirmed improvement or deterioration (95% CI), weeks**	**n***	**Cumulative % by week 36 (95% CI)**	**Median time to confirmed improvement or deterioration (95% CI), weeks**		
FACT-G FWB	159	25.1 (17.3, 33.6)	NE (86.1, NE)	78	31.1 (17.5, 45.7)	NE (NE, NE)	0.87 (0.47, 1.61)	0.665
**FACT-LeuS**	159	23.0 (15.2, 31.7)	NE (NE, NE)	79	29.2 (16.9, 42.6)	NE (49.6, NE)	0.68 (0.37, 1.26)	0.223
**FACT-Leu TOI**	159	23.4 (15.8, 31.9)	NE (NE, NE)	79	34.8 (20.1, 49.9)	NE (26.6, NE)	0.70 (0.38, 1.27)	0.241
**FACT-G TS**	159	34.1 (25.0, 43.4)	NE (38.9, NE)	79	41.1 (25.4, 56.1)	NE (15.1, NE)	0.80 (0.46, 1.36)	0.403
**FACT-Leu TS**	159	25.9 (17.7, 34.8)	NE (NE, NE)	79	34.3 (20.2, 48.8)	NE (25.6, NE)	0.69 (0.38, 1.24)	0.215
EQ-5D-5L								
UK-based HUI	159	27.2 (18.7, 36.4)	NE (NE, NE)	79	23.2 (13.4, 34.6)	NE (49.6, NE)	0.93 (0.50, 1.72)	0.821
US-based HUI	159	17.1 (10.8, 24.8)	NE (105.6, NE)	79	25.5 (15.0, 37.4)	NE (NE, NE)	0.66 (0.35, 1.25)	0.200
**EQ-VAS**	158	17.9 (11.4, 25.7)	NE (NE, NE)	79	31.0 (17.9, 45.0)	NE (36.1, NE)	0.51‡ (0.27, 0.96)	0.036

Domains/subscales in bold are primary domains of interest. Percentages are Kaplan-Meier estimates of the proportion of patients with confirmed improvement or deterioration at 36 weeks.

CI, confidence interval; EQ-VAS, EuroQol visual analogue scale; EWB, emotional well-being; FACT-G, Functional Assessment of Cancer Therapy – General; FACT-Leu, Functional Assessment of Cancer Therapy – Leukemia; FWB, functional well-being; HUI, health utility index; HR, hazard ratio; LeuS, leukemia “additional concerns” subscale; NE, not estimable; PRO, patient-reported outcome; PWB, physical well-being; SWB, social/family well-being; TOI, trial outcome index; TS, total score.

*Number of patients in the PRO-evaluable population who were at risk of the event of interest at baseline.

†HR (95% CI) and nominal p values for ponatinib versus imatinib were calculated using a stratified Cox proportional hazards regression model adjusting for the randomization age strata (≥18 to <45, ≥45 to <60, ≥60 years).

‡Nominal $P < 0.05$.

Supplemental Figure 1. Patient disposition. *Patients who discontinued treatment but were still in follow-up. EOT, end of treatment; HSCT, hematopoietic stem cell transplantation; ITT, intent-to-treat; PRO, patient-reported outcome.

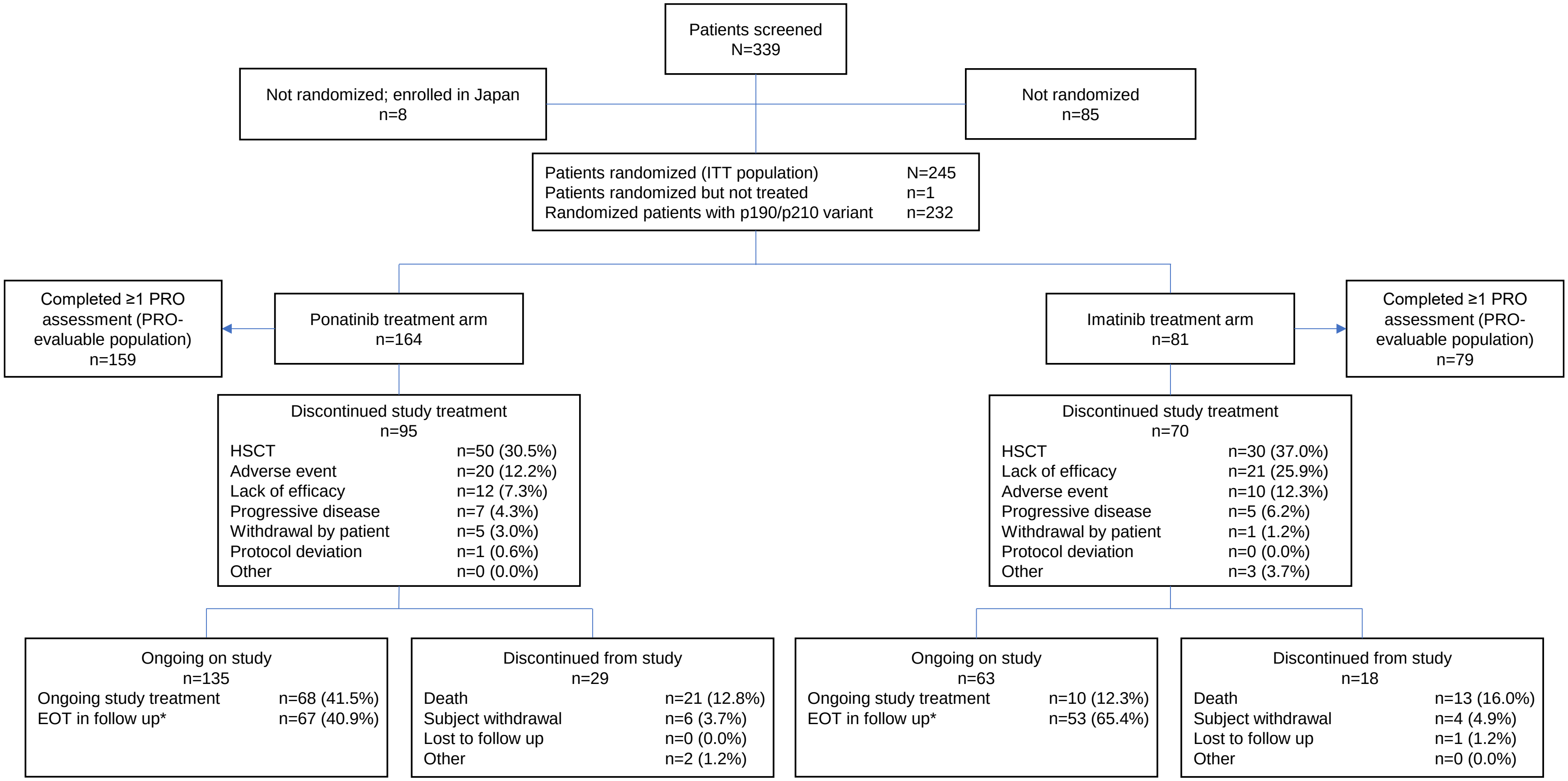

Supplemental Figure 2. Proportions of patients in the ITT population eligible and ineligible for PRO assessments over time. “Other reasons” included ineligibility due to death, which occurred in a total of 5/164 (3.0%) patients in the ponatinib arm and 1/81 (1.2%) patients in the imatinib arm. C, cycle; CR, complete remission; D, day; HSCT, hematopoietic stem cell transplantation; ITT, intent-to-treat; MRD, minimal residual disease; PD, progressive disease; PRO, patient-reported outcome.

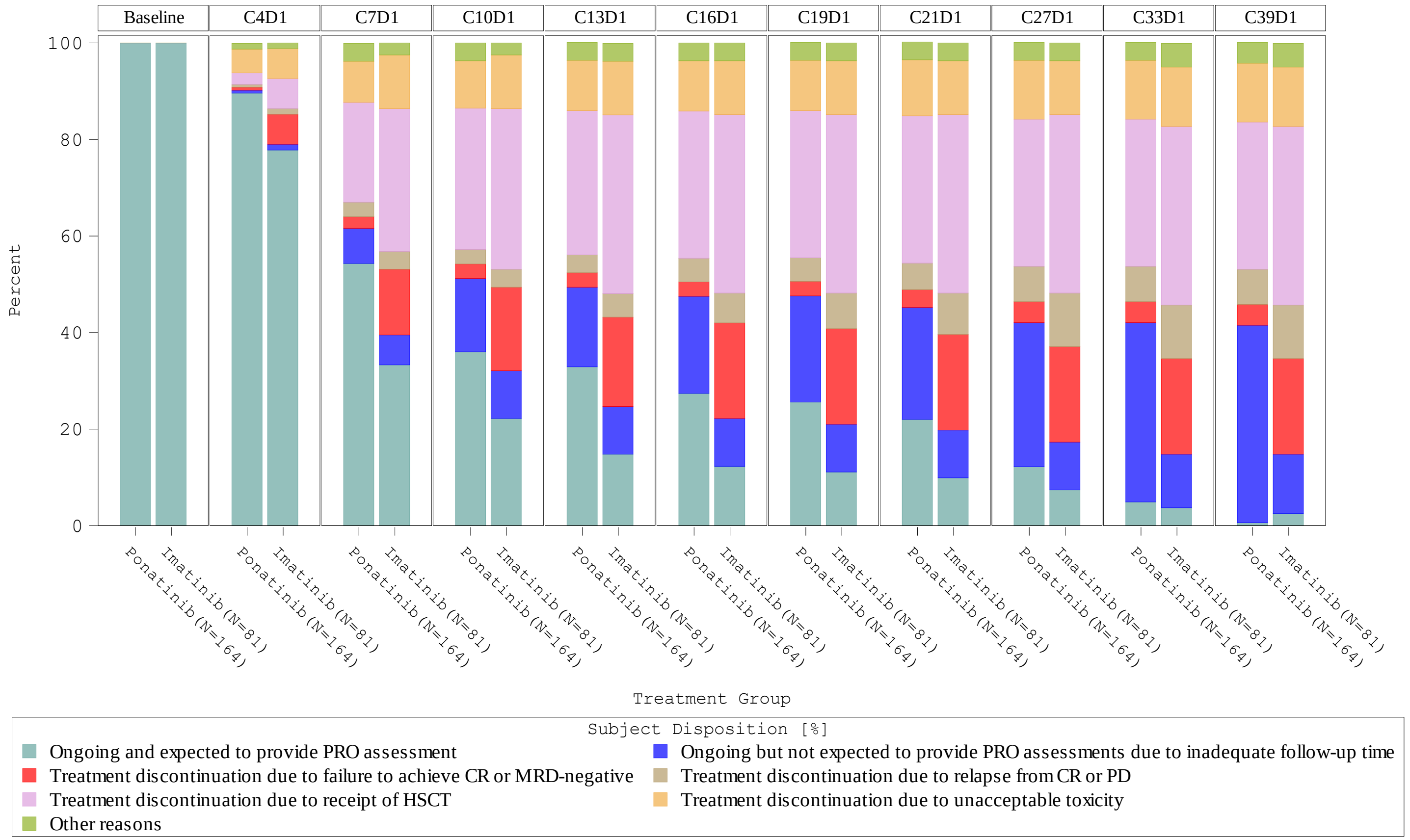

Supplemental Figure 3. Distribution of responses for FACT-GP5 (PRO-evaluable population). The FACT-GP5 item asks patients to respond to the question "I am bothered by side effects of treatment" using a five-point scale from 0 ("Not at all") to 4 ("Very much"). Numbers below the bars represent the numbers of patients available for assessment at each visit. Data labels represent the percentage of patients in each category. C, cycle; D, day; EOT, end of treatment; FACT-GP5, Functional Assessment of Cancer Therapy – General Item GP5; Ima, imatinib; Pon, ponatinib; PRO, patient-reported outcome.

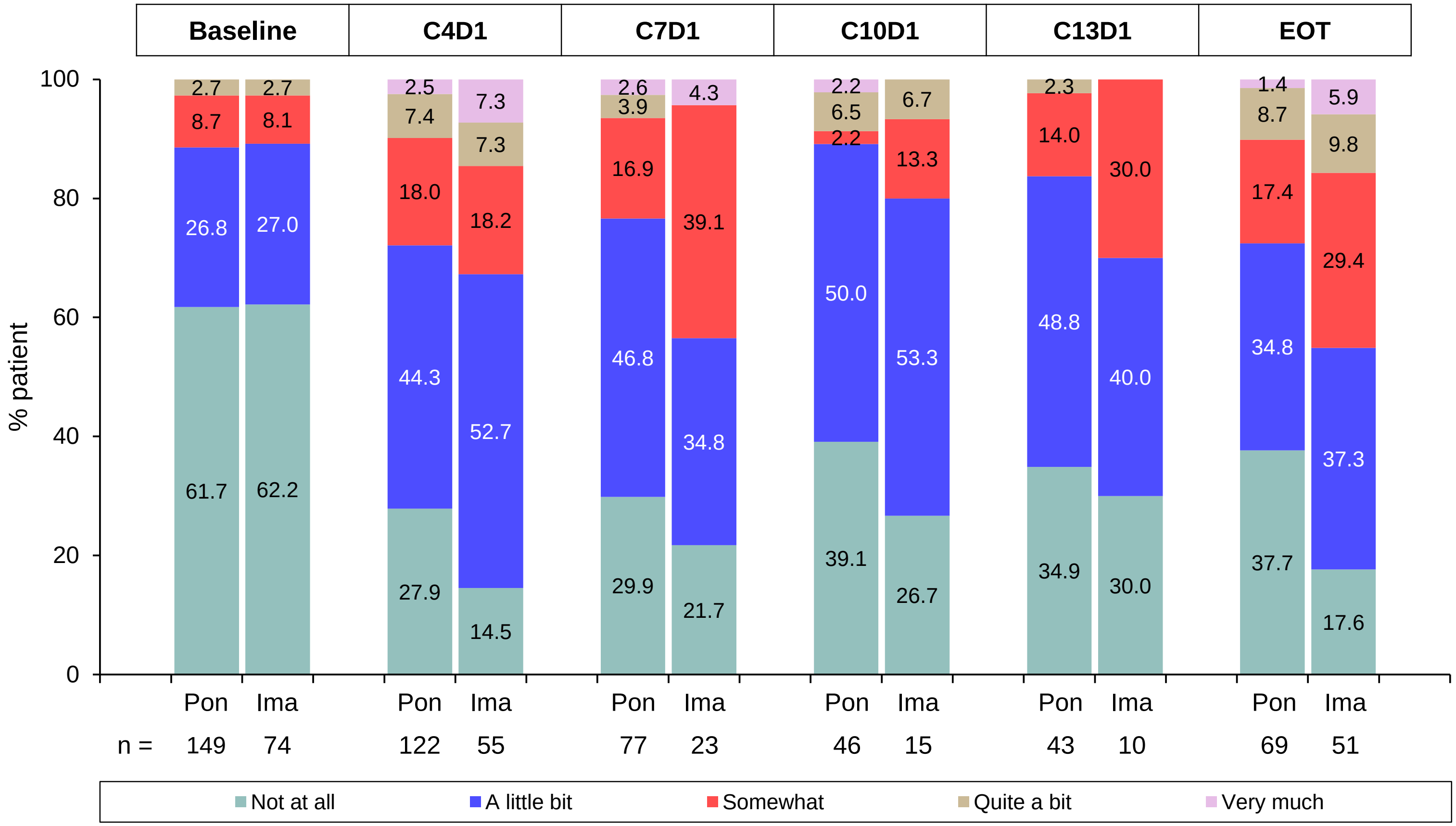

Supplemental Figure 4. Distribution of changes from baseline in response level for FACT-GP5 (PRO-evaluable population). The FACT-GP5 item asks patients to respond to the question “I am bothered by side effects of treatment” using a five-point scale from 0 (“Not at all”) to 4 (“Very much”). Numbers below the bars represent the numbers of patients available for assessment at each visit. Data labels represent the percentage of patients in each category. C, cycle; D, day; EOT, end of treatment; FACT-GP5, Functional Assessment of Cancer Therapy – General Item GP5; Ima, imatinib; Pon, ponatinib; PRO, patient-reported outcome.

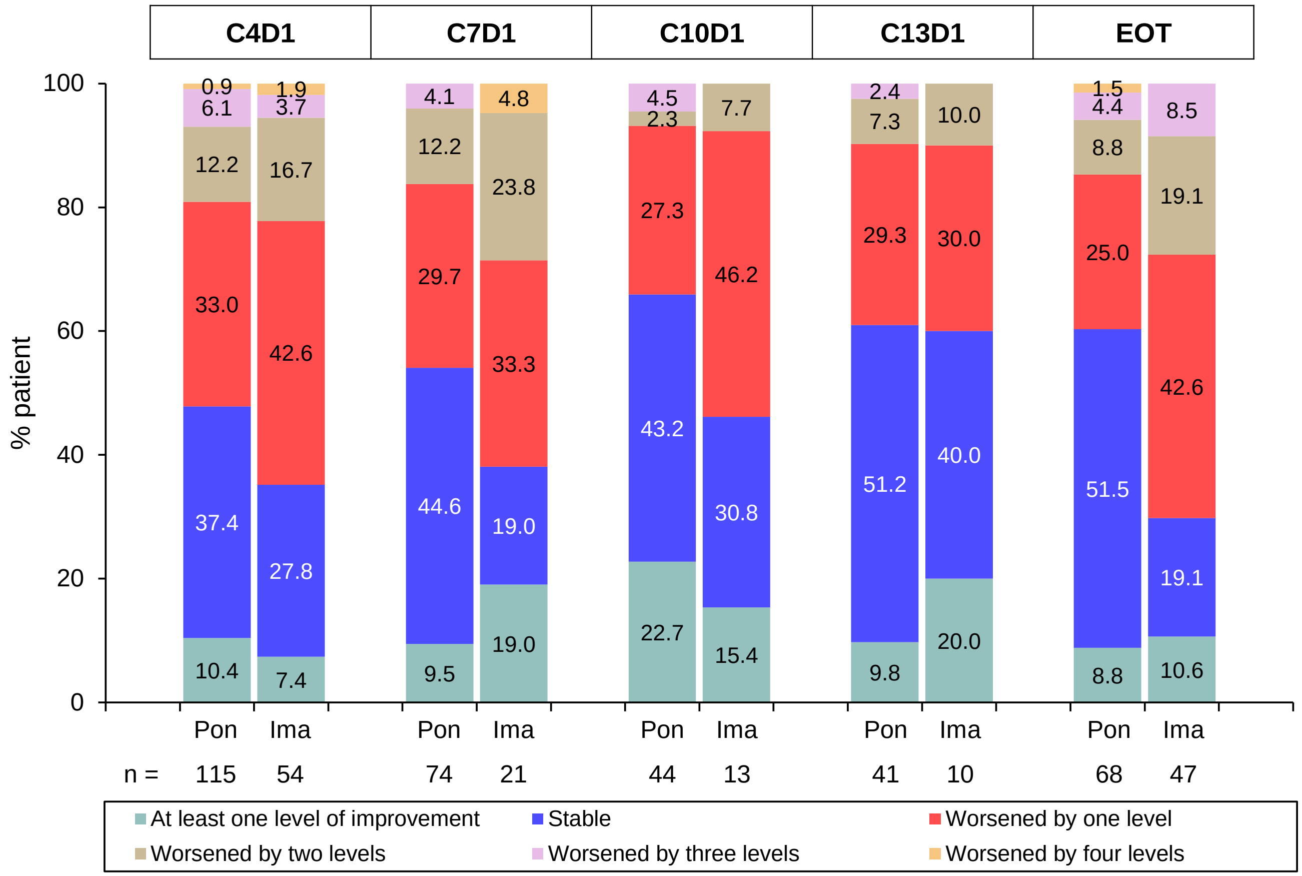

Appendix. PhALLCON Institutional Review Board/Independent Ethics Committees

Advarra, Inc
6940 Columbia Gateway Dr
Suite 110
Columbia, MD 21046
UNITED STATES

Comissao Nacional de Etica em Pesquisa - CONEP
SRTV 701, Via W 5 Norte, Lote D
Edificio PO 700, 3 Andar, Asa N
Brasilia/DF 70719-040
BRAZIL

Comitato Etico Brianza
Via Pergolesi, 33
Monza (MB) 20090
ITALY

Comite d'ethique de la Recherche du CIUSSS de l'Est-de-I'lle-de-Montreal
5415 Boul Assomption
Montreal, Quebec H1T 2M4
CANADA

Comite de Etica de la Investigacion con medicamentos del Hospital Universitari Germans Trias y Pujol
Crta. de Canyet. s/n
Badalona, Barcelona 08916
SPAIN

Comite de Protection des Personnes - CPP Ouest II
Maison de la Recherche Clinique
CHU Angers
4, rue Larrey
Angers Cedex 9 49933
FRANCE

Ethics Committee for Clinical Trials at Ministry of Health
8, Damyan Gruev Str.
Sofia, Sofia 1303
BULGARIA

Ethics Committee of Federal State Budgetary Institution
“National Medical Research Centre named after V.A. Almazov” of the Ministry of Health of the Russian Federation
21, Kolomyazhsky prospect
Saint Petersburg 197341
RUSSIA

Ethics Committee of Federal State Budgetary Institution
"National Medical Research Centre of Haematology" of the Ministry of Health of the Russian Federation
4, Noviy Zykovskiy Proezd
Moscow 125167
RUSSIA

Ethikkommission des Landes Oberosterreich
Kepler Universitatsklinikum, Neuromed Campus
Wagner-Jauregg Weg 15
Linz, Upper Austria 4020
AUSTRIA

HUS Tutkimuseettiset toimikunnat
Biomedicum Helsinki 2 C, 7.krs
Tukholmankatu 8 C
Helsinki 00018
FINLAND

Monash Health Human Research Ethics Committee
Level 2, I Block, Monash Medical Centre
246 Clayton Rd
Clayton, Victoria 3168
AUSTRALIA

National Cancer Center IRB#2-J
5-1-1, Tsukiji
Chuo-ku, Tokyo 104-0045
JAPAN

National Ethics Committee
284 Mesogeion Ave
Cholargos, Attica 15562
GREECE

Niezalezna Komisja Bioetyczna ds. Badan Naukowych przy Gdanskim Uniwersytecie Medycznym
Debinki 7, Gdansk
Pomorskie 80-211
POLAND

Ottawa Health Science Network Research Ethics Board
Civic Campus, Box 675
725 Parkdale Ave
Ottawa, Ontario K1Y 4E9
CANADA

UBC BC Cancer Research Ethics Board
Fairmont Medical Bldg
750 W Broadway, Suite 1315
Vancouver, BC V5Z 1J3
CANADA

WCG Institutional Review Board
1019 39th Ave SE, Suite 120
Puyallup, WA 98374
UNITED STATES

Franciscan Health Institutional Review Board EST
8111 S Emerson Ave, Suite 208
Indianapolis, IN 46237
UNITED STATES

Comitato Etico ASL Lecce
Via Miglietta, 5
Lecce, Lecce 73100
ITALY

Comite de Etica en Investigacion del Hospital Universitario
"Dr. Jose Eleuterio Gonzalez"
Ave Francisco I. Madero y Ave Gonzalitos s/n, Col Mitras Centro
Monterrey, Nuevo Leon 64460
MEXICO

Comite de Etica em Pesquisa da Universidade de Passo Fundo
Pro-Reitoria de Pesquisa e Pos-Graduacao - VRPPG/UPF
Km 292, BR 285, Campus I, Bairro Sao Jose
Passo Fundo/Rio Grande do Sul (RS) 99052-900
BRAZIL

Comite de Etica em Pesquisa do Hospital Sao Rafael
Ave Sao Rafael, 2152
Salvador/BA 41253-190
BRAZIL

Ankara Universitesi Tip Fakultesi Klinik Arastirmalar Etik Kurulu
Ankara Universitesi Tip Fakultesi Morfoloji Binasi
Sihhiye, Ankara 06100
TURKEY

IRB of Okayama University Hospital
2-5-1 Shikata-cho
Kita-ku, Okayama-city
Okayama, 700-8558
JAPAN

Research Ethics Committee
China Medical University Hospital
2 Yude Rd, Taichung 40447
TAIWAN

Comite Institucional de Etica de Investigacion en Salud del Hospital Privado
Centro Medico de Cordoba Ave Naciones Unidas 346 B
Parque Velez Sarsfield
Cordoba, X5016KEH
ARGENTINA

Comitato Etico per la Sperimentazione Clinica (CESC) ULSS 3 Serenissima
Nucleo per la Ricerca Clinica AULSS 3
Via Don Federico Tosatto, 14
Mestre Venezia 30174
ITALY

Comitato Etico Regionale Della Liguria
Largo Rosanna Benzi, 10
Genova, Genova 16132
ITALY

General Hospital of Athens "Evangelismos", Scientific Council
45-47 Ipsilantou str
Athens, Attica, 10676
GREECE

Comite de Etica em Pesquisa do Hospital Erasto Gaertner
Liga Paranaense de Combate ao Cancer
Rua Doutor Ovande do Amaral, 201
Curitiba/PR 81520-060
BRAZIL

Institution Review Board
National Cheng Kung University Hospital
138 Sheng-Li Rd
Tainan 704
TAIWAN

Comitato Etico dell'Universita Sapienza
Viale del Policlinico, 155
Roma, Roma 00161
ITALY

Comitato Etico IRCCS Ospedale San Raffaele
Via Olgettina, 60
Milano, Milano20132
ITALY

Comitato Etico Palermo 2, Segreteria Scientifica
Azienda Ospedaliera Ospedali Riuniti Villa Sofia Cervello
Polo Ospedaliero Cervello
Via Trabucco 180
Palermo, Palermo 90146
ITALY

Comite de Etica em Pesquisa do Hospital de Clinicas de Porto Alegre da Universidade Federal do Rio Grande do Sul - HCPA/UFRGS
Ave Protasio Alves, 211
Portao 4 - 5 andar do Bloco C
Rio Branco, Porto Alegre/RS 90410-000
BRAZIL

IRB of The First Hospital of Jilin University
No 1 Xinmin St, Changchun
Jilin Province, 130021
CHINA

Biomedical Research Alliance of New York, LLC/IRB
1981 Marcus Ave, Suite 210
Lake Success, New York 11042
UNITED STATES

Ethikkommission der Medizinischen Universitat Wien
Borschkegasse 8b/E06
Vienna 1090
AUSTRIA

ACTIVATO Joint IRB
1-1, Seiryo-machi, Aoba-ku
Sendai-city, Miyagi 980-8574
JAPAN

Comitato Etico dell’Area Vasta Emilia Nord
Via del Pozzo, 71
Modena, Modena 41124
ITALY

Comite Institucional de Etica de Investigacion en salud del Sanatorio Allende
Ave Hipolito Yrigoyen 384
Cordoba, X5000JHQ
ARGENTINA

Clinical Research Ethics Committee of The First Affiliated Hospital of Zhejiang University School of Medicine
No 79, Qingchun Rd, Hangzhou
Zhejiang, 310003
CHINA

Inje University Haeundae Paik Hospital
Institution Review Board
875 Haeundaero
Haeundae-gu, Busan 48108
SOUTH KOREA

The Catholic University of Korea, St. Vincent's Hospital
Institutional Review Board
93, Jungbu-daero
Paldal-gu, 16247
SOUTH KOREA

Yeungnam University Hospital Institution Review Board
170, Hyeonchung-ro,
Nam-gu, Daegu, 42415
SOUTH KOREA

Aiiku Hospital Institutional Review Board
2-1, S4W25, Chuo-ku
Sapporo, Hokkaido 064-0804
JAPAN

Local Ethics Committee at State Autonomous Healthcare Institution os Sverdlovsk "Sverdlovsk Regional Clinical Hospital #1"
185, Volgogradskaya St
Ekaterinburg, Sverdlovsk 620102
RUSSIA

Chinbuk National University Hospital
Institution Review Board
20 Geonji-ro, Deokjin-gu
Jeonju-si, Jeollabuk-do 54907
SOUTH KOREA

General Hospital of Thessaloniki G. Papanikolaou
Scientific Council
Papanikolaou Ave
Thessaloniki, Exochi/Asvestochori 57010
GREECE

Comitato Etico della Romagna (CEROM)
IRST IRCCS s.r.l.
Via Piero Maroncelli, 40
Meldola, 47014
ITALY

Comitato Etico Indipendente di Area Vasta Emilia Centro
c/o Segreteria locale
Via Albertoni, 15
Bologna, Bologna 40138
ITALY

OHSU Institutional Review Board
3181 SW Sam Jackson Park Rd
Portland, Oregon 97239
UNITED STATES

Research Ethics Committee Hualien Tzu Chi Hospital, Buddhist Tzu Chi Medical Foundation
707, Sec.3, Chung-Yang Rd
Hualien 97002
TAIWAN

Comite de Etica em Pesquisa do Hospital Amaral Carvalho
Fundaçao Doutor Amaral Carvalho
Rua das Palmeiras, 89
Jau/SP, 17210-120
BRAZIL

Comite de Etica em Pesquisa do Instituto Estadual de Hematologia Arthur de Siqueira Cavalcani - HEMORIO
Rua Frei Caneca, 8
Rio de Janeiro/RJ 20211-030
BRAZIL

Tokai University Hospital Institutional Review Board
143 Shimokasuya
Isehara City, Kanagawa 259-1193
JAPAN

Comite de Etica em Pesquisa da Faculdade de Ciencias Medicas da Universidade Estadual de Campinas - UNICAMP
Rua Tessalia Vieira de Camargo, 126 - Cidade Universitária Profesor Zeferino Vaz Distrito de Barao Geraldo
Campinas/SP 13083-887
BRAZIL

University General Hospital of Heraklion, Scientific Council
Crossroads of Vouton-Stavrakion
Heraklion, Crete71110
GREECE

“Attikon” University General Hospital, Scientific Council
1 Rimini St
Chaidari, Attica 12462
GREECE

Comite de Etica em Pesquisa do Hospital das Clinicas da Faculdade de Medicina de Ribeirao Preto da USP - HCFMRP/USP
Campus Universitario S/N - Monte Alegre
Ribeirao Preto/SP 14048-900
BRAZIL

Medical Ethics Committee of The First Affiliated Hospital of Soochow University
No 899 Pinghai St, Suzhou
Jiangsu, 215006
CHINA

Comissao de Etica para Analise de Projetos de Pesquisa do HCFMUSP
Rua Ovidio Pires de Campos, 225
5 andar
Predio da Administracao, Cerqueira Cesar
Sao Paulo/SP 05403-010
BRAZIL

Comite de Etica em Pesquisa da Fundacao Antonio Prudente
A.C. Camargo Cancer Center
Rua Professor Antonio Prudente, 211
Sao Paulo/SP 01509-010/01509-900
BRAZIL

Local Ethic Committee of Moscow
State Clinical Hospital named after V.V. Veresaev
10, Lobnenskaya St
Moscow 127644
RUSSIA

Fondazione Policlinico Universitario A. Gemelli IRCCS
c/o Segreteria tecnico Scientifica
L.go A. Gemelli 8
00168 Roma – RM
ITALY

Ethikkommission der Stadt Wien
Thomas-Klestil-Platz 8/2, TownTown
Wien, 1030
AUSTRIA

Kyungpook National University Hospital Institutional Review Board
130 Dongduk-ro
Jung-gu, Daegu 41944
SOUTH KOREA

University General Hospital of Patras "Panagia I Voithia"
Scientific Council
Rio, Patra, Achaia 26504
GREECE

University General Hospital of Larissa, Scientific Council
Mezourlo, Larissa, Thessaly, 41110
GREECE

Roswell Park Institutional Review Board
Elm and Carlton Streets
Buffalo, NY 14263
UNITED STATES

Ethics Committee of Institute of Hematology & Blood Diseases Hospital, Chinese Academy of Medical Sciences
No 288 Nanjing Rd, Heping District
Tianjin, 300041
CHINA

IRB of Henan Cancer Hospital
No 127 Dongming Rd
Zhengzhou, Henan 450008
China

Institutional Review Board of Chiba Aoba Municipal Hospital
1273-2 Aobacho, Chuo-ku
Chiba-shi, Chiba 260-0852
JAPAN

UCLA Office of the Human Research Protection Program
10889 Wilshire Blvd, Suite 830
Los Angeles, California 90095
UNITED STATES
